# Expression of *ZjPSY*, a Phytoene Synthase Gene from *Zoysia japonica* Affects Plant Height and Photosynthetic Pigment Contents

**DOI:** 10.3390/plants11030395

**Published:** 2022-01-31

**Authors:** Di Dong, Yuhong Zhao, Ke Teng, Penghui Tan, Zhuocheng Liu, Zhuoxiong Yang, Liebao Han, Yuehui Chao

**Affiliations:** 1 School of Grassland Science, Beijing Forestry University, Beijing 100083, China; didoscori@163.com (D.D.); tengke@grass-env.com (K.T.); liuzhuocheng@bjfu.edu.cn (Z.L.); yangzhuoxiong@bjfu.edu.cn (Z.Y.); 2 Animal Science College, Tibet Agriculture & Animal Husbandry University, Nyingchi 860000, China; zhaoyh@xza.edu.cn; 3 Beijing Academy of Agriculture and Forestry Sciences, Beijing 100083, China; 4 Beijing Chaoyang Foreign Language School, Beijing 100101, China; penghuitan@bjfu.edu.cn

**Keywords:** carotenoids, dwarfing, *Zoysia japonica*

## Abstract

Phytoene synthase (PSY) is a key limiting enzyme in the carotenoid biosynthesis pathway for regulating phytoene synthesis. In this study, *ZjPSY* was isolated and identified from *Zoysia japonica*, an important lawn grass species. *ZjPSY* cDNA was 1230 bp in length, corresponding to 409 amino acids. *ZjPSY* showed higher expression in young leaves and was downregulated after GA_3_, ABA, SA, and MeJA treatments, exhibiting a sensitivity to plant hormones. Regulatory elements of light and plant hormone were found in the upstream of *ZjPSY* CDS. Expression of *ZjPSY* in *Arabidopsis thaliana* protein led to carotenoid accumulation and altered expression of genes involved in the carotenoid pathway. Under no-treatment condition, salt treatment, and drought treatment, transgenic plants exhibited yellowing, dwarfing phenotypes. The carotenoid content of transgenic plants was significantly higher than that of wild-type under salt stress and no-treatment condition. Yeast two-hybrid screening identified a novel interacting partner *ZjJ2* (*DNAJ homologue 2*), which encodes heat-shock protein 40 (HSP40). Taken together, this study suggested that ZjPSY may affect plant height and play an important role in carotenoid synthesis. These results broadened the understanding of carotenoid synthesis pathways and laid a foundation for the exploration and utilization of the *PSY* gene.

## 1. Introduction

In plants, carotenoids are important isoprenoid compounds, which play classical roles in photosynthetic biological processes, including photomorphogenesis and photoprotection. Carotenoids also participate in plant color formation as pigments, which affect plant color through different levels of aggregation in the chromoplasts [1]. Carotenoids range in color from colorless to yellow and red and are reflected in the fruits and leaves of many plants [2]. In addition, carotenoids and their oxidative and enzymatic lysates are considered to be signaling molecules for interactions of plants with environment, so carotenoids are proposed to play an important regulatory role in plant growth and development [3]. 

**Figure 1:**
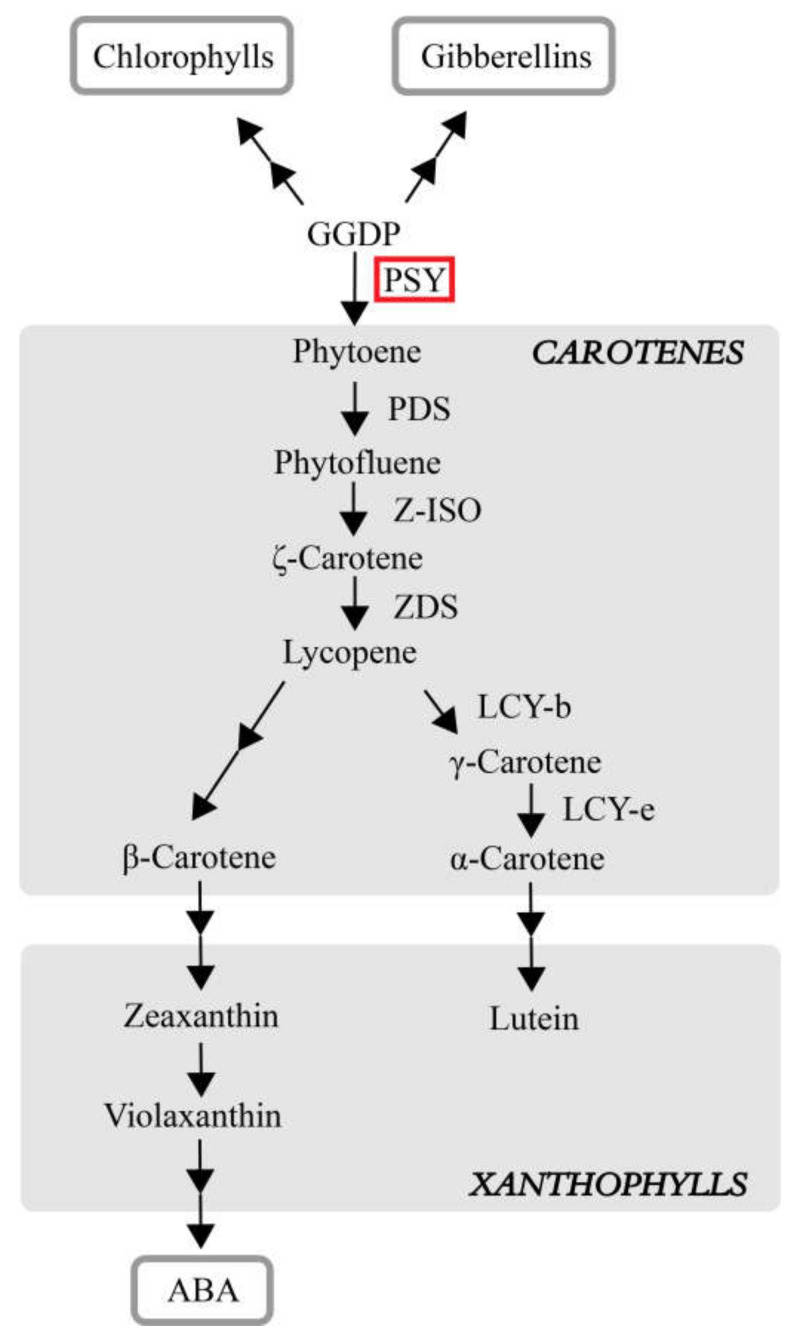
*PSY* expression regulates the synthesis of carotenoids and is involved in the synthesis of hormones [4,5,6,7]. PDS, phytoene desaturase. Z-ISO, 15-cis-ζ-carotene isomerase. ZDS, ζ-carotene desaturase. LCY-b, Lycopene β-cyclase. LCY-e, lycopene ε-cyclase.

Phytoene is a precursor for the synthesis of all carotenoid substances. *Phytoene synthase* (*PSY*), a key enzyme regulating carotenoid synthesis, can catalyze the conversion of geranylgeranyl pyrophosphate (GGPP) to phytoene, which is the first rate-limiting reaction in the carotenoid synthesis pathway (Figure 1) [4,5,6,7]. GGPP also acts as a precursor for gibberellin, abscisic acid (ABA), and chlorophylls [4].

*PSY* is a preferred candidate gene for understanding the molecular regulation of carotenoid accumulation in most species [8]. Constitutive PSY overexpression could increase total carotenoid and β-carotene contents [2,9]. Transgenic tobacco (*Nicotiana benthamiana*) with overexpression of *PSY* significantly increased carotenoid content, and the *PSY* gene showed a key role in lycopene accumulation in transgenic autumn olive fruits (*Elaeagnus umbellata*) [9,10]. The *PSY* genes contain several isoforms that show tissue-specific expression. There is only one *PSY* gene in *Arabidopsis thaliana*, and overexpression of endogenous *PSY* leads to delayed seed germination and increased carotenoid and chlorophyll levels [11]. There are two PSY genes reported in carrots (*Daucus carota*) and three in tomato (*Solanum lycopersicum*) and rice (*Oryza sativa*) [12,13,14]. *SlPSY1* is required for carotenoid synthesis in tomato fruits, and *SlPSY2* is necessary for carotenoid synthesis in leaf tissues, whereas *SlPSY3* is related to root resistance under stress [15]. The *PSY* gene may also be involved in plant dwarfing; expression of *PSY* from *Oncidium* Gower Ramsey in Tobacco showed dwarfing and reduced leaf area phenotypes [16]. The study of the *PSY* gene is beneficial to deepening the understanding of basic metabolism and regulation of the carotenoid synthesis pathway. 

*Zoysia japonica* is a common type of warm-season turfgrass. Due to its resistance to drought and salt, it has been widely used in golf courses, sports grounds, and urban greening. Although the genes involved in the carotenoid biosynthesis pathway have been identified, the regulation of these genes in plant growth, development, and stress tolerance is not fully understood. Although the carotenoid synthesis pathway has been confirmed to be critical in plant growth regulation [1,2,3], the regulation mode of carotenoid accumulation and the regulation mechanism of the *PSY* gene in *Zoysia japonica* have not been studied. In addition, dwarfing is an important index in the production and application of turfgrass. Dwarfing can improve planting density, enhance photosynthetic efficiency and reduce pruning rate, thus improving the quality of turfgrass [17]. In this study, the *PSY* gene of *Zoysia japonica* was isolated and identified for a biological and functional study, which could lead to further insight into the function of the *PSY* gene and lay a foundation for studying the role of carotene in Zoysia.

## 2. Results

### 2.1. Identification and Bioinformatic Analysis of the ZjPSY Gene in Zoysia japonica

According to the Zoysia Genome Database [18], the DNA fragment containing *ZjPSY* coding domain sequences (CDSs) was cloned and ligated into cloning vector pMD19-T. The ZjPSY cDNA sequences were deposited in the NCBI database with accession numbers KY264127.1. The full-length *ZjPSY* cDNA contains a 1230 bp ORF encoding a protein of 409 amino acids which belongs to the Isoprenoid_Biosyn_C1 superfamily. According to the Compute pI/MW tool of Expasy, the molecular weight of ZjPSY was 46.39 kD, and the theoretical isoelectric point was 9.20. Intron/exon organization analysis showed that *ZjPSY* had five exons (Figure S1A). Secondary structure analysis indicates that ZjPSY has 63% alpha helix, 1% beta strand, and 25% disorder proteins (Figure S1B). A 3D Tertiary structure model of ZjPSY provides insights into its molecular geometry (Figure S1C). The stereochemical structure supported the α-helical nature of the enzyme.

**Figure 2:**
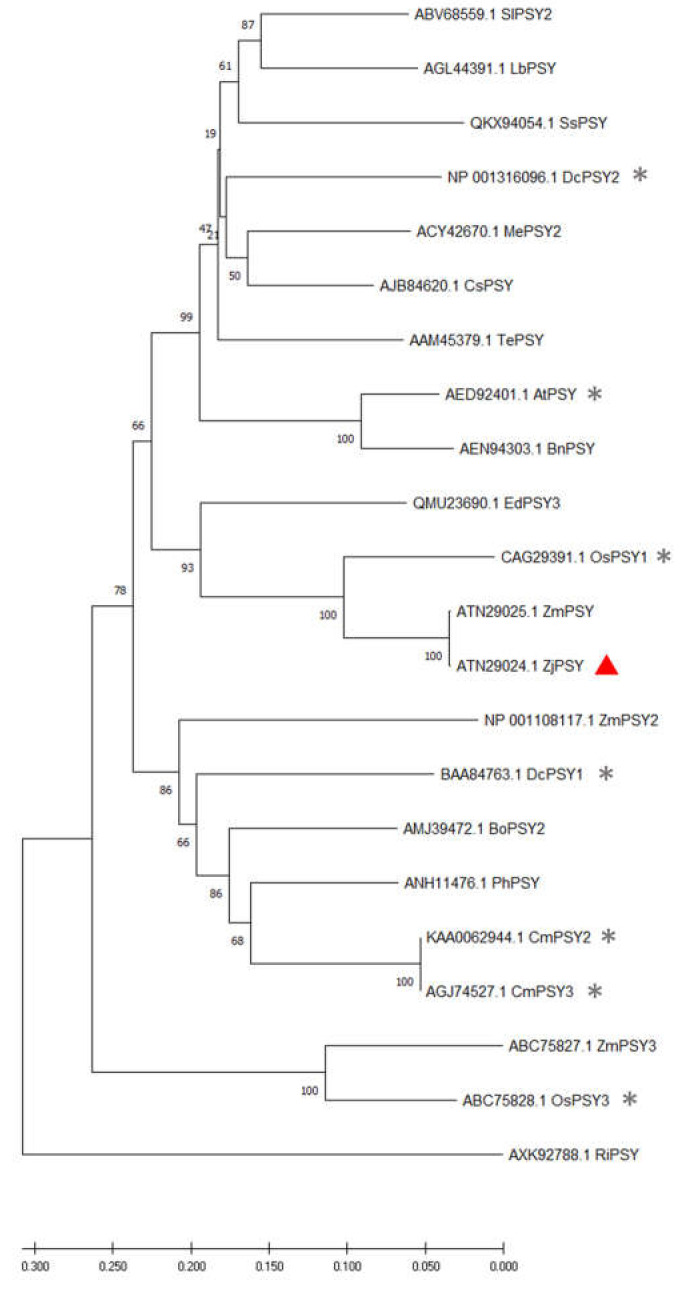
Phylogenetic tree of PSY. Phylogenetic tree analysis among 22 PSY proteins. Protein sequence accession numbers are as follows: SlPSY2 (ABV68559.1), *Solanum lycopersicum*; IbPSY (AGL44391.1) [19], *Ipomoea batatas*; SsPSY (QKX94054.1), *Salvia splendens*; MePSY2 (ACY42670.1), *Manihot esculenta*; CsPSY (AJB84620.1) [20], *Camellia sinensis*; DcPSY2 (NP_001316096.1) [21], *Daucus carota*; TePSY (AAM45379.1), *Tagetes erecta*; AtPSY (AED92401.1) [22], *Arabidopsis thaliana*; BnPSY (AEN94303.1), *Brassica napus*; EdPSY3 (QMU23690.1), *Eleocharis dulcis*; OsPYS1 (CAG29391.1) [23], *Oryza sativa*; ZmPSY (ATN29025.1), *Zoysia matrella*; ZjPSY (ATN29024.1), *Zoysia japonica*; ZmPSY2 (NP_001108117.1), *Zea mays*; DcPSY1 (BAA84763.1) [21], *Daucus carota*; CmPSY2 (KAA0062944.1) [24], *Cucumis melo*; CmPSY3 (AGJ74527.1) [24], *Cucumis melo*; BoPSY2 (AMJ39472.1), *Bixa orellana*; PhPSY (ANH11476.1), *Prunus humilis*; RiPSY (AXK92788.1), *Rubus idaeus*; ZmPSY3 (ABC75827.1), *Zea mays*; OsPSY3 (ABC75828.1) [25], *Oryza sativa*. ▲ Mark represented the ZjPSY. * Represents the PSY protein was analyzed.

**Figure 3:**
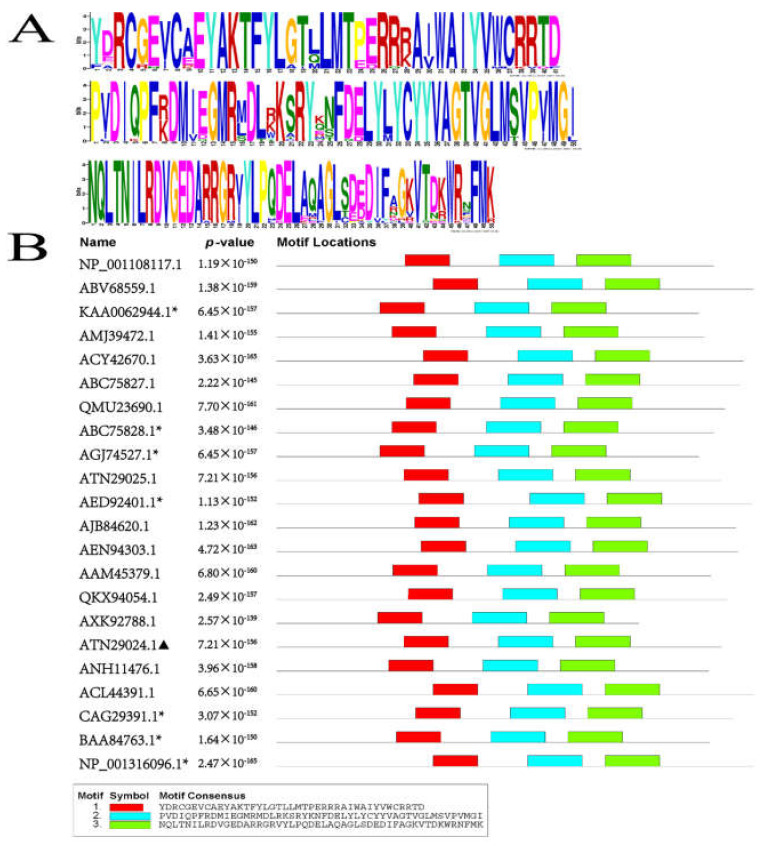
Conserved structure analysis of PSYs. (**A**) The conserved motifs in 22 PSY proteins of different species. Top scoring three motifs consisting of amino acids composition are listed. (**B**) Protein motif analyses of PSY protein sequence. Each color shows the different conserved motif structure identified in the PSY protein. ▲ Mark represented the ZjPSY. * Represents the PSY protein has been analyzed.

Phylogenetic analysis of PSY proteins was performed based on the protein sequences from different species collected from NCBI database, with a neighbor-joining method (Figure 2). Phylogenetic analysis of the PSY protein revealed that ZjPSY has the highest homology with PSY of *Zoysia matrella*, though it was most closely related to OsPSY1 of *Oryza sativa*. To further dissect the homology of PSY, the proteins in 22 different species were further analyzed for the occurrence of conserved motifs. Motif sequence scan analysis showed the top three scored motifs with presence of amino-acid rich profiles (Figure 3A). There were distinct patterns indicating that the three motifs were found in all PSY protein sequences (Figure 3B). The three motifs were found to be highly conserved. In this respect, the phylogenetic analysis and motif analysis revealed that ZjPSY had a low similarity to PSY3 from *Oryza sativa* and *Zea mays* and PSY from *Rubus idaeus*.

### 2.2. Prediction of Cis-Regulatory Elements of ZjPSY Promoter

**Figure 4:**
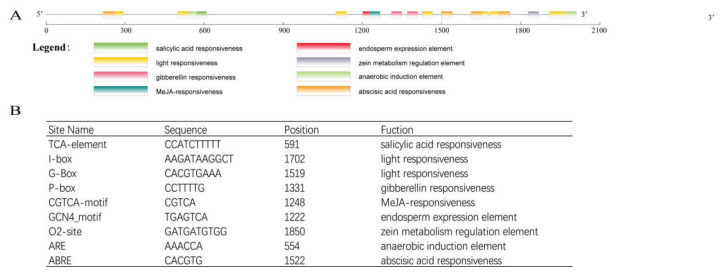
Analysis of up-stream sequence of *ZjPSY* gene. (**A**) Cis-acting elements upstream of *ZjPSY* gene. (**B**) List of predicted binding sites for the transcription factors upstream of *ZjPSY* gene.

To analyze the regulatory pathway and function of ZjPSY, a 2000 bp upstream sequence from the translation start site (ATG) was examined using the PlantCARE database (Figure 4) [26]. Multiple light responsive motifs, including I-box and G-box, were identified in the frequency of occurrence of cis-elements. In addition, hormone response elements, such as gibberellin responsiveness P-box, salicylic acid responsiveness TCA-element, MeJA-responsiveness TGACG-motif, and CGTCA-motif were also identified. 

In addition, several cis-acting elements involved growth and development were found, such as endosperm expression element GCN4_motif and zein metabolism regulation element O2-site. 

### 2.3. ZjPSY Was Localized in the Chloroplast and Cytoplasm 

**Figure 5:**
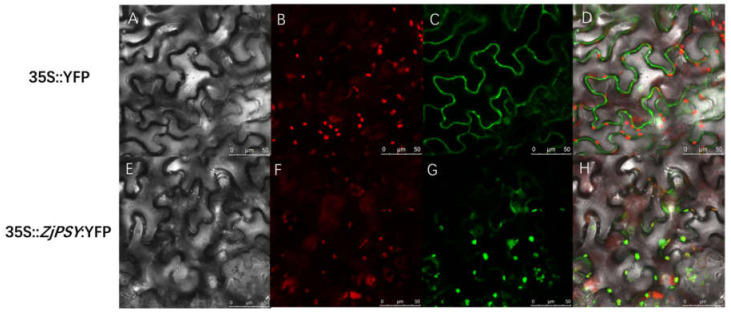
Subcellular localization of ZjPSY in plant cells. (**A**–**D**) 35S::YFP fluorescent detection. (**E**–**H**) 35S::ZjPSY:YFP fluorescent detection. (**A**,**E**): bright light; (**B**,**F**): chlorophyll autofluorescence; (**C**) and (**G**): YFP fluorescence; (**D**,**H**): merged signal.

We next sought to examine the subcellular localization of ZjPSY proteins in tobacco leaf via transient transformation. The untargeted YFP was transfected as a control and the fluorescence signal localized in the nucleus and cytoplasm (Figure 5A–D). In the leaf tissue which transiently expressed YFP fusion proteins (35S::*ZjPSY*:YFP), YFP fluorescence signal colocalized with the autofluorescence of chlorophyll in the chloroplast. The fluorescence accumulated in the chloroplast and cytoplasm (Figure 5E–H), suggesting that ZjPSY mainly localized in chloroplast and cytoplasm. 

### 2.4. ZjPSY Interacts with DNAJ Homologue 2

**Figure 6:**
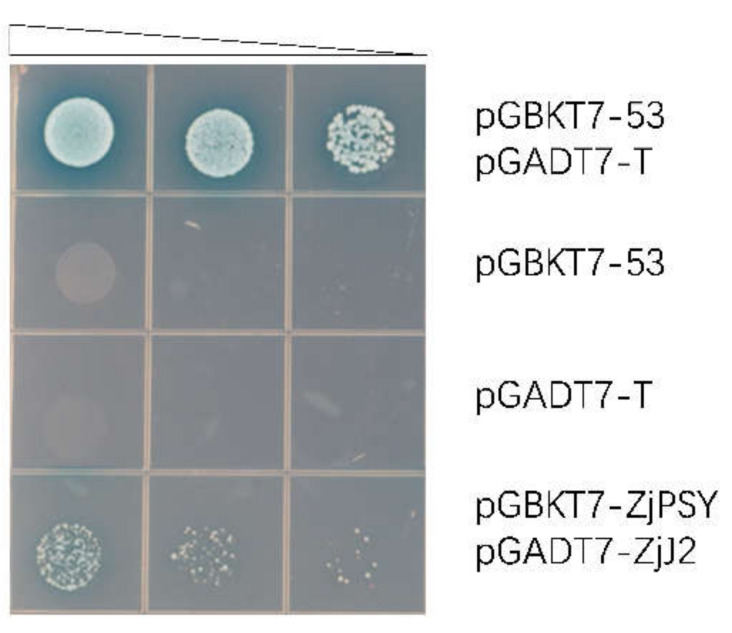
Screening for ZjPSY interacting proteins with yeast two-hybrid assay. Interaction between *ZjJ2* and *ZjPSY*. Yeast cells were spotted on higher stringency QDO/X/A agar plates by 10-fold serial dilutions. Blue clones were positive and white or absent clones were negative. pGBKT7-53 or pGADT7-T was used as the negative control, and pGBKT7-53 and pGADT7-T were co-transformed as positive control.

To analyze the protein interaction of ZjPSY, yeast two-hybrid assays and Bimolecular Fluorescence Complementation (BiFC) assays were performed. The *J2* gene in Zoysia was identified, and it had high homology with *J2* in Arabidopsis. In Arabidopsis, *J2* encodes heat-shock protein 40 (HSP40) [27,28]. The results showed yeast cells with pGADT7-*ZjJ2* and pGBKT7-*ZjPSY* grew well and turned blue on QDO/X/A plates, similar to the positive control (Figure 6). These results indicate that ZjJ2 protein interact with ZjPSY protein. 

**Figure 7:**
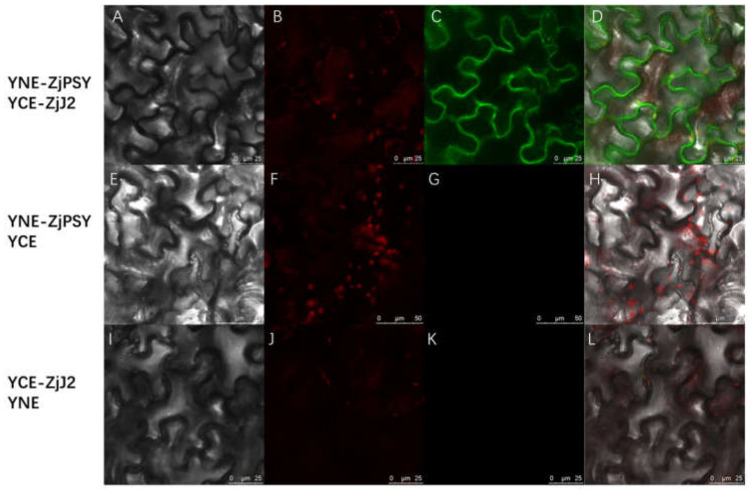
The interaction between ZjPSY and ZjJ2 in living cells shown by BiFC. (**A**–**D**) YNE-ZjPSY and YCE-ZjJ2 fluorescent detection. (**E**–**H**) YNE-ZjPSY and YCE fluorescent detection. (**I**–**L**) YCE-ZjJ2 and YNE fluorescent detection. (**A**,**E**,**I**): Bright light; (**B**,**F**,**J**): chlorophyll autofluorescence; (**C**,**G**,**K**): YFP fluorescence; (**D**,**H**,**L**): merged signal. Co-expression of YNE-ZjPSY with YCE or YCE-ZjJ2 with YNE was used as negative control.

BiFC was used to confirm the interaction of ZjPSY and ZjJ2 in plant cells. ZjPSY and ZjJ2 were fused with the C and N terminals of YFP, respectively, and transiently expressed in tobacco cells. When YNE-ZjPSY and YCE-ZjJ2 were co-expressed, the YFP fluorescence was captured (Figure 7A–D), while no YFP signal was found in negative samples (Figure 7E–L). Those results demonstrated and confirmed the interaction of ZjPSY with ZjJ2. 

### 2.5. Expression Pattern of ZjPSY 

**Figure 8:**
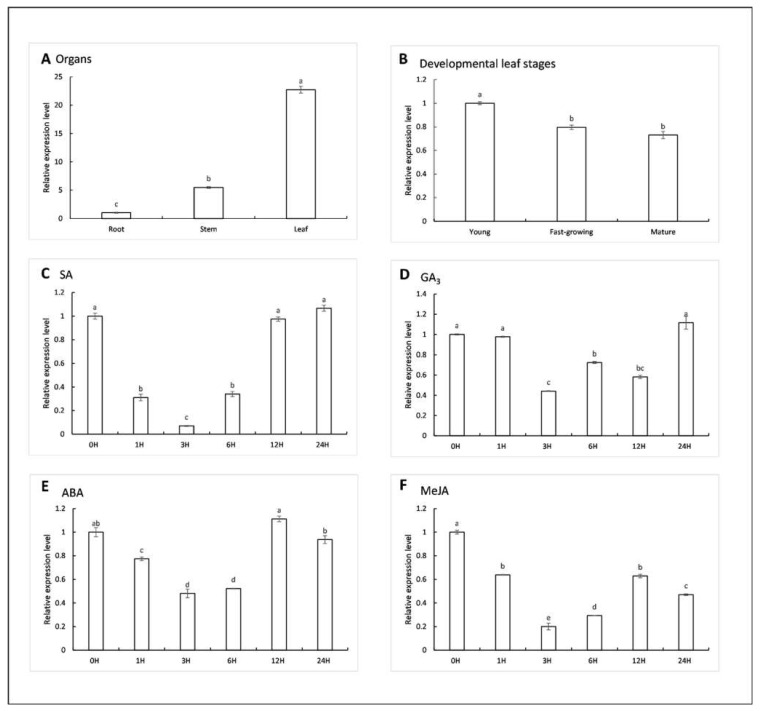
Expression profiles of *ZjPSY*. (**A**) *ZjPSY* expression pattern in root, stem, and leaf. (**B**) *ZjPSY* expression pattern in leaves at different developmental stages. (**C**–**F**) Expression pattern in leaf under 10 μM GA_3_ treatment (**C**), 10 μM ABA treatment (**D**), 0.5 mM SA treatment (**E**), and 10 μM MeJA treatment (**F**). Different letters above the columns indicate significant differences (*p* ≤ 0.05, *n* = 3). *ZjPSY* expression in root (**A**), young stage (**B**), and 0 h (**C**–**F**) was set as calibrator sample with a relative expression equal to 1.

Real-time PCR was conducted to examine the expression pattern of *ZjPSY* in different tissues and development stages. The expression profile results showed that *ZjPSY* was detected in root, stem, and leaf tissues, and the expression was significantly abundant in leaf (Figure 8A). *ZjPSY* expression was relatively high in early leaf growth stage and slightly decreased in fast-growing and mature leaves (Figure 8B). The expression levels of *ZjPSY* were downregulated by multiple hormone treatments, including GA_3_, ABA, SA, and MeJA (Figure 8C–F). After leaves were treated with these hormones for 3 h, the expression of ZjPSY reached the lowest level at 3 h. The expression patterns suggested that *ZjPSY* may be involved in leaf development and hormone responses. 

### 2.6. Chlorophyll and Carotenoid Contents in Transgenic Plants

**Figure 9:**
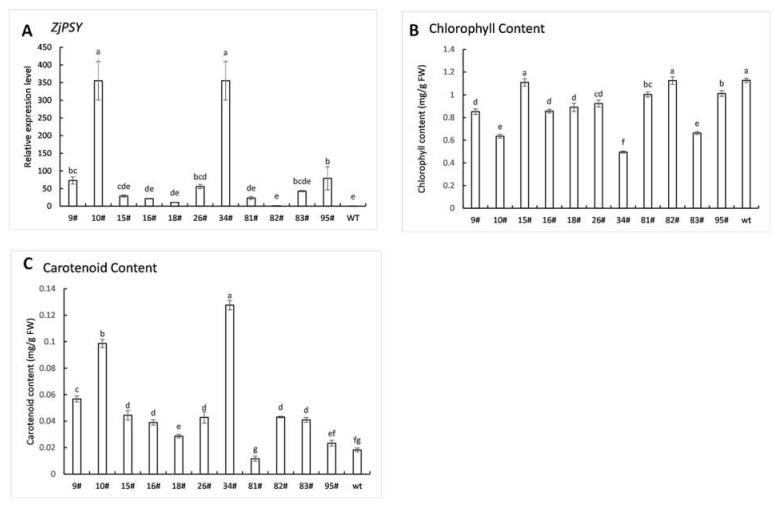
(**A**) The qRT-PCR analysis of ZjPSY expression in wild-type plants and the transgenic lines. Transgenic line designated #82 was set as calibrator sample with a relative expression equal to 1. (**B**) Chlorophyll contents of wild-type plants and the transgenic lines. (**C**) Carotenoid contents of wild-type plants and the transgenic lines. Different letters above the columns indicate significant differences (*p* ≤ 0.05).

To investigate whether *ZjPSY* expression affects chlorophyll and carotenoid contents in the plant, genetic transformation was conducted using the Agrobacterium-mediated method. Expression levels in different lines were measured by qRT-PCR, and most transgenic lines had much higher expression levels, indicating the *ZjPSY* gene was successfully expressed in transgenic Arabidopsis (Figure 9A). To explore the effect of *ZjPSY*, chlorophyll and carotenoid contents were detected in transgenic lines and wild-type plants (Figure 9B,C). The lines with the high *ZjPSY* expression levels enhanced carotenoid levels and decreased chlorophyll levels, which indicates that expression of *ZjPSY* affects carotenoid and chlorophyll contents.

### 2.7. ZjPSY Expression in Arabidopsis Thaliana Resulted in Leaf Yellowing and Plant Dwarfing

**Figure 10:**
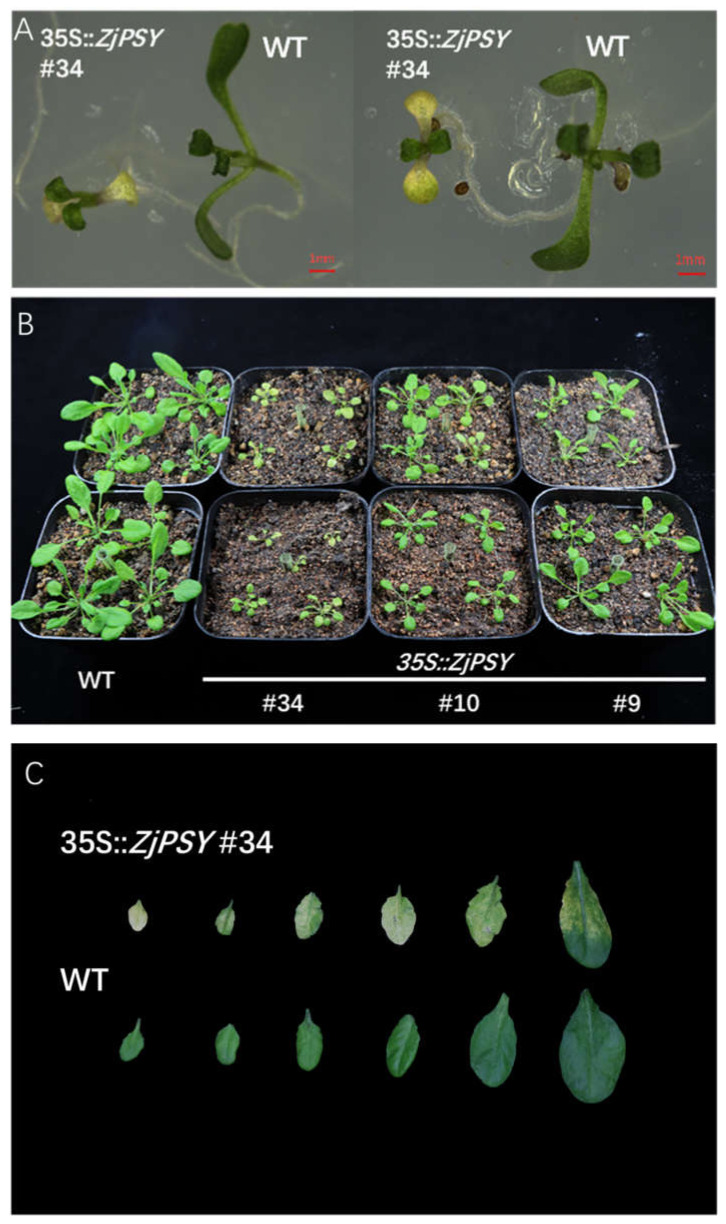
Leaf yellowing and dwarfing phenotype of transgenic Arabidopsis expressing the *ZjPSY* gene and wild-type Arabidopsis. (**A**) Transgenic Arabidopsis #34 expressing *ZjPSY* gene and wild-type Arabidopsis of 5 days. (**B**) Comparison of the phenotypes of transgenic Arabidopsis and wild-type Arabidopsis at 30 days. (**C**) Typical leaves in WT and transgenic Arabidopsis at 45 days.

Based on the expression levels and carotenoid and chlorophyll contents, three positive transgenic lines designated #9, #10, #34 were selected for further study of the *ZjPSY* gene. Grown on MS plates, transgenic seedlings had etiolated cotyledons, showing *ZjPSY* decreased chlorophyll contents in early stages of transgenic plants (Figure 10A). Grown in soil, transgenic plants displayed dwarf phenotype and senescent leaves compared with the WT (Figure 10B,C). These results suggest that *ZjPSY* may be involved in leaf pigment accumulation and plant senescence. 

### 2.8. Regulation of Plant Growth, Plant Pigment, and Pigment-Related Gene Expression by ZjPSY 

**Figure 11:**
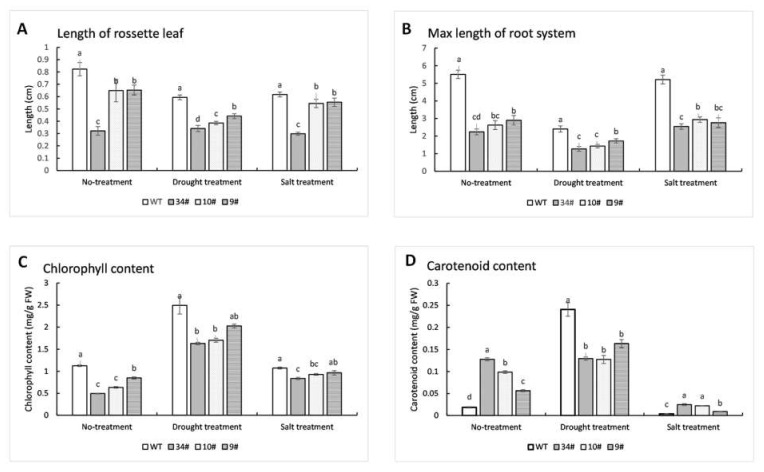
Statistical analysis of plant height and chlorophyll and carotenoid contents in transgenic and WT Arabidopsis. The transgenic and wild-type Arabidopsis grown in MS agar plates for 14 days: 200 mM Nacl and 500 mM mannitol were added to MS medium in drought treatment and salt treatment groups, respectively. (**A**,**B**) Length of rosette leaf (**A**) and maximum length of root system (**B**) in transgenic lines and wild-type. Different letters above the columns indicate significant differences (*p* ≤ 0.05, *n* = 20). (**C**,**D**) Chlorophyll (**C**) and carotenoid (**D**) contents in transgenic lines and wild-type Arabidopsis. Different letters above the columns indicate significant differences (*p* ≤ 0.05, *n* = 3).

To further detect the transgenic plants, the three selected transgenic lines and the wild-type were transplanted into MS medium (Figure S2). The rosette leaf lengths and taproot lengths of the three transgenic lines were significantly smaller than WT plants, especially in the transgenic line (#34) with higher *ZjPSY* expression levels (Figure S2, Figure 11A,B). According to statistical analysis, the transgenic plants in the line #34 had 39.01% smaller rosette leaf length and 46.03% smaller taproot length, compared with wild-type (Figure 11A,B). The chlorophyll content of transgenic lines was significantly lower than that of WT plants, while the carotenoid content of transgenic lines was significantly higher than that of WT plants (Figure 11C,D). These results indicated that expression of *ZjPSY* in *Arabidopsis thaliana* significantly increased carotenoid contents and decreased chlorophyll contents. 

Stresses can affect plant pigment contents. Different lines were placed on MS agar plates containing 200 mM mannitol for drought treatment or 100 mM NaCl for salt stress (Figure S2B,C). Similar to no-treatment conditions, the plant height of MS transgenic lines was significantly lower than WT lines after mannitol or NaCl was added (Figure S2B,C, Figure 11A,B). Under drought treatment and salt treatment for 14 days, transgenic lines showed an obvious dwarfing phenotype with root lengths and rosette leaf lengths significantly lower than those of wild-type. Under drought treatment, the carotenoid content of the transgenic plants did not show similar performance to that of no treatment; the increase in carotenoid content of transgenic plants was much lower than that of wild-type (Figure 11D). The chlorophyll content of transgenic plants #34 and #10 was lower than that of wild-type, but the chlorophyll content of transgenic plant #9 was not significantly different from that of wild-type (Figure 11C). Under salt treatment, the chlorophyll contents of transgenic plants #34 and #10 were lower than those in wild-type (Figure 11C), and the carotenoid level of the transgenic plants was higher than that of the wild-type (Figure 11D). The results suggests that expression of *ZjPSY* may be involved in inhibiting carotenoid accumulation or accelerating its degradation under drought treatment. In plants subjected to salt stress, transgenic plants with *ZjPSY* gene overexpression showed higher levels of carotenoids than WT plants. 

**Figure 12:**
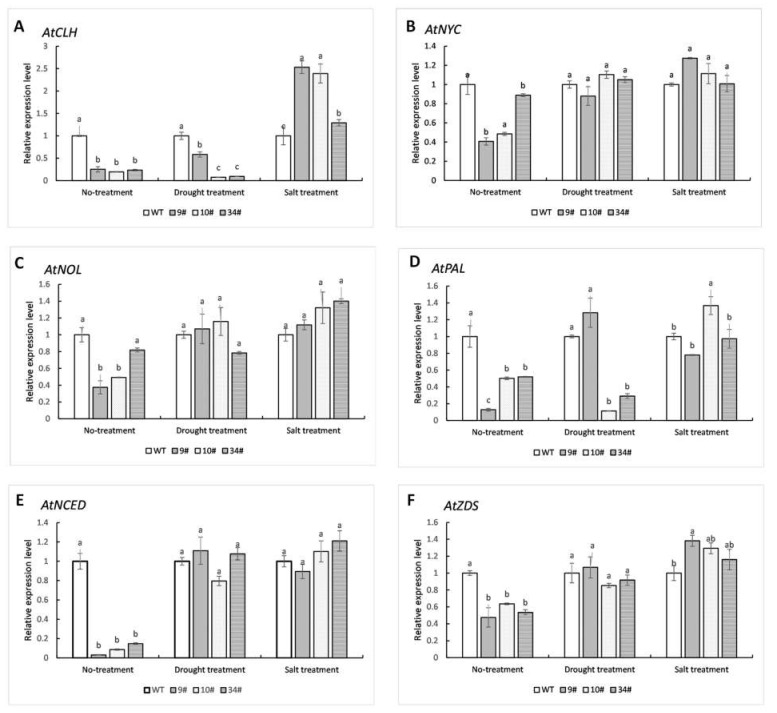
Pigment-related gene expression level of wild-type and transgenic plants subjected to no-treatment, drought, and salt stress conditions. Pigment-related genes include *AtCLH* (**A**), *AtNYC* (**B**), *AtNOL* (**C**), *AtPAL* (**D**), *AtNCED* (**E**), and *AtZDS* (**F**). WT in no-treatment, drought treatment, or salt treatment was set as calibrator sample with a relative expression equal to 1.

To gain more insight into the role of *ZjPSY* in plant growth and development, the expression profiles of six pigment-genes, *AtCLH (Chlorophyllase)*, *AtNYC* *(NON-YELLOWCOLORING)*, *AtNOL (NYC1-LIKE)*, *AtPAL (Phenylalanine ammonia-lyase)*, *AtNCED (9-cis-epoxycarotenoid dioxygenase)*, and *AtZDS* *(Z-carotene desaturase)*, were determined using qRT-PCR (Figure 12). *AtCLH* catalyzes the hydrolysis of ester bonds to chlorophyllide and phytol, which is the first step of chlorophyll degradation [29]. *AtNYC* and *AtNOL* are involved in chlorophyll band light-harvesting complex II degradation [30,31,32]. *AtPAL*, a key enzyme in the phenylalanine pathway, catalyzes the deamination of phenylalanine into trans-cinnamic acid, which is related to anthocyanin synthesis [33]. *AtNCED*, a kind of carotenoid cleavage enzyme, is a key enzyme that regulates ABA biosynthesis under stress [34]. *AtZDS* plays a regulatory role in the catalysis of Z-carotene to tetra-cis lycopene and is also an important gene in the production of carotenoids [35]. Under no-treatment conditions, the relative expression levels of several pigment-related genes measured in transgenic plants were lower than those in wild-type. Expect for *AtCLH* and *AtPAL*, there was no significant difference in the relative expression levels of other measured genes between wild-type and transgenic plants under drought treatment. In salt stress, *AtCLH* was significantly increased comparing with WT, and the relative expression of *AtZDS* was also slightly increased. Salt treatment may not influence the expression of *AtNYC*, *AtNOL*, *AtPAL*, or *AtNCED*.

## 3. Discussion

The *PSY* gene regulates the first committed step in the process of carotenoid biosynthesis. In this study, the *ZjPSY* gene was cloned from *Zoysia japonica*. Bioinformatics analysis showed that the open reading frame of the *ZjPSY* gene was 1230 bp, encoding 409 amino acids. The protein belongs to the isoprenoid_biosyn_C1 superfamily. The large central cavity formed by the antiparallel α helixes and the two aspartic acid rich regions in the opposite wall constitute its catalytic sites. Structural analysis shows that ZjPSY contains many α helixes, and its three-dimensional analog structure contains a large central cavity (Figure S1C). Evolutionarily conserved motifs in different species revealed similar functions [36], and three motifs were found in 22 PSY expressed species. Phylogenetic analysis of PSY proteins with a neighbor-joining method revealed that ZjPSY is also more homologous with OsPSY1 and OsPSY3 in Oryza sativa. In addition, ZjPSY had a low similarity to PSY in model plant *Arabidopsis thaliana*.

According to the prediction of regulatory elements, the ZjPSY promoter is expected to mainly include the following cis-acting sequences: TCA-element, I-box, G-box, P-box, CGTCA-motif, GCN4-motif, O2-site, ARE, and ABRE. The regulatory element analysis of the *ZjPSY* promoter contains of a variety of hormone responsive elements. TCA Element, P-box, TGACG Motif, and ABRE element are involved in responsiveness to salicylic acid, gibberellin, MeJA, and ABA, respectively [37,38,39,40]. The G-Box is involved in light responsiveness. Transcription factor *HY5* (*LONG HYPOCOTYL 5*) and *PIFs* (*PHYTOCHROME INTERACTING FACTORS*), which are regulators of chlorophyll and carotenoid biosynthesis, impart regulation to PSY through direct binding to the G-box cis-element in *Arabidopsis thaliana* [41]. Based on the cis-element analysis of the *ZjPSY* promoter, we further explored the expression pattern of *ZjPSY* using qRT-PCR. The expression pattern results suggest that *ZjPSY* was abundant in developing leaves. *ZjPSY* might be highly sensitive to hormone signaling; it was significantly downregulated under GA_3_, ABA, SA, and MeJA treatment. These results indicated it was involved in pathways of different hormone signal response. 

To investigate the functions of *ZjPSY* in plants, the 35S::*ZjPSY*:YFP was constructed and transformed to *Arabidopsis thaliana* through the floral dip method. The transgenic plants showed yellowing and dwarfing phenotypes. *OncPSY* expression has also contributed to the dwarfing of tobacco [16].The dwarfed phenotype in transgenic plants may be caused by the conversion of large amounts of GGPP to phytoene due to the increase in active PSY protein, which leads to the suppression of the gibberellin synthesis pathway (Figure 1) [42]. 

**Figure 13:**
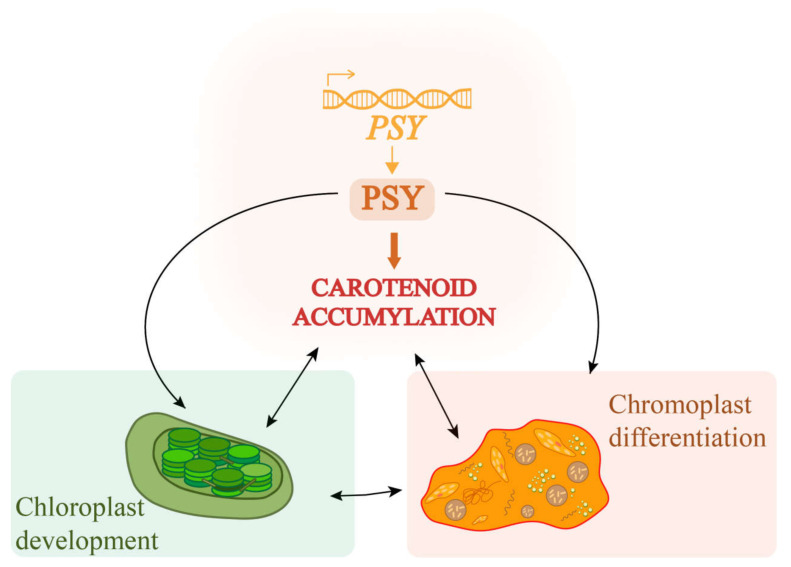
Molecular schematic of PSY’s involvement in carotene accumulation and chloroplast development in plants [1]. The development of chloroplasts and chromoplast differentiation are related to PSY activity and sink capacity for carotenoids.

The color of carotenoids in photosynthetic tissues is usually masked by chlorophyll, and carotenoids could provide bright coloration characteristics in tissue with low chlorophyll content [43]. The content of chlorophyll and carotenoids in wild-type and transgenic plants were determined. Compared with wild-type, carotenoid content of transgenic plants increased significantly, and chlorophyll content decreased significantly under no treatment, which indicated *ZjPSY* not only changed leaf color by increasing the accumulation of carotenoids but also affected chlorophyll. Studies have shown that plants adapt to photoprotective pigments by regulating the development of plastid structures. *PSY* is involved in the coordination process of carotenoid biosynthesis and storage with the molecular factors of photosynthetic development by participating in carotenogenesis (Figure 13) [44]. The ZjPSY was localized specifically to the chloroplast and cytoplasm consistent with the function of carotenogenesis and involvement of chloroplast development. 

In order to further explore the effect of *ZjPSY* on plant leaf color, the expression levels of six pigment-related genes, *AtCLH*, *AtNYC*, *AtNOL*, *AtPAL*, *AtNCED*, and *AtZDS*, were assessed. Under no-treatment conditions, the expression levels of six genes were lower than those of wild-type plants. Under drought treatment, the relative expression levels of most genes had no significant difference except *AtCLH* and *AtPAL*. However, under salt treatment, the expression pattern of *AtCLH* was completely opposite to the no-treatment condition. Correspondingly, the amount of carotenoid and chlorophyll synthesis was different under different treatments. Expression of *ZjPSY* could increase carotenoid content under no-treatment conditions and salt treatment but decreased under drought treatment. Expression of *LbPSY* in *Escherichia coli* and yeast cells could improve tolerance in high salinity and drought environment [44]. Studies on *Daucus carota* have confirmed that *DcPSY* participates in ABA-mediated salt stress tolerance through the binding of promoters and ABRE transcription factors [45]. In this experiment, the transgenic plants showed no significant enhanced stress tolerance and even showed premature senescence under drought treatment. Due to the low homology of PSY between model plants and *Zoysia japonica*, it may be caused by differences in PSY between species. Copy volume and expression level of *PSY* in different transgenic lines also affects the phenotype of the transgenic plants [44]. 

In this study, yeast two-hybrid screening identified *J2* as a novel interacting partner of *ZjPSY*. The *J2* gene encodes heat-shock protein 40 (HSP40) isoforms J2, a molecular chaperone that helps to prevent proteins from misfolding [27]. DnaJ-like chaperone has been shown to be involved in carotenoid synthesis in several species. Expression of Orange protein that belongs to DnaJ-Like chaperones in *Arabidopsis thaliana* can increase the abundance of active PSY protein, but the transcription level of the *PSY* gene does not change. It was inferred to be the chief posttranscriptional regulator of PSY in carotenoid biosynthesis [46]. Expression of gene for orange protein increased lutein and β-carotene in *Chlamydomonas reinhardtii* [47]. DnaJ-like chaperone is considered to support phytoene synthase [43]. In addition, experiments have shown that defective farnesylation of HSP40 is sufficient to induce ABA hypersensitivity [5]. It is possible that *PSY* and *J2* work together by affecting ABA activity in plants. The pathway of carotenoid synthesis greatly affects the endogenous ABA levels [5]. In this study, *ZjJ2* was confirmed as a novel interactive partner of *ZjPSY*, but its interaction mode needs further experimental verification.

In summary, ZjPSY may be involved in plant development and pigment synthesis. In response to abiotic stress, overexpression of ZjPSY may have different regulatory effects under no-treatment condition, salt stress, and under drought stress. However, the molecular mechanisms need to be further studied.

## 4. Materials and Methods

### 4.1. Plant Materials and Growth Conditions 

*Zoysia japonica* cultivar ‘Compadre’ seeds were purchased from the Hancock seed company (Hancock, FL, USA). *Zoysia japonica* was cultivated in pots, kept at 28/23 °C (day/night) with 16 h (at 400 mmol/m^2^/s)/8 h photoperiod and 50% humidity. All the Arabidopsis materials used in this study were of a Col ecotype background. The Arabidopsis seeds were sown on Murashige and Skoog plates (4.43 g/L Murashige and Skoog powder, 8 g/L agar, pH 5.8), with a 16 h white light (at 90 mmol/m2/s)/8 h dark cycle and 50% humidity at 25 °C. After the fourth leaves appeared on the seedlings, the seedlings were transferred to sterilized soil or culture medium to continue growth under the same growth conditions.

### 4.2. Identification and Cloning of ZjPSY 

Total RNA was isolated from ‘Compadre’ leaves of 3-month-old samples using Plant RNA Kit (Omega Bio-tek, Atlanta, USA), and the first-strand cDNA was synthesized using the PrimeScriptTM II 1st Strand cDNA Synthesis Kit (TAKARA, Dalian, China). Based on the sequence of *ZjPSY* (Zjn_sc00008.1.g00200.1.sm.mkhc) in the Zoysia Genome Database [18], primer sequences for cloning were designed, and PCR products were ligated into the cloning vector pDM19-T for storage and further experiments. All primers used in this study are listed in Table S1. 

### 4.3. Bioinformatics Analysis

BLAST analysis of the NCBI database was used to identify homologs, and 22 PSY protein sequences from other species were obtained. The phylogenetic tree and ZjPSY gDNA structure were constructed using MEGA version 6.0 with the neighbor-joining method and GSDS 2.0 (http://gsds.gao-lab.org/Gsds_about.php (accessed on 6 October 2021)) [48]. The molecular weights and theoretical isoelectric points were analyzed by Compute pI/MW tool (http://web.expasy.org/compute_pi/ (accessed on 15 September 2021)). The motif analysis of PSY amino acids and cis-regulatory elements in the promoter involved the use of MEME (http://meme-suite.org/tools/meme (accessed on 15 September 2021)), PlantCARE database (http://bioinformatics.psb.ugent.be/webtools/plantcare/html/ (accessed on 15 September 2021)) and TBtools [49,50]. The ZjPSY protein model was generated by using the Phyre v2.0 tool (www.sbg.bio.ic.ac.uk/phyre2/ (accessed on 6 October 2021)).

### 4.4. Vector Construction and Generation of Transgenic Plants

The completed coding region of *ZjPSY* was amplified by PCR with primers ZjPSY-F and ZjPSY-R (Table S1). The fragment containing the complete CDS was ligated to the vector plasmid pMD19-T and stored in *E. coli* for long-term preservation. The 35S::*ZjPSY*:YFP was constructed by fusing completed *ZjPSY* CDS followed by YFP with primers, 3302Y-ZjPSY-F and 3302Y-ZjPSY-R. The recombination was introduced into *Agrobacterium tumefaciens* GV3101, which was then used for the transformation of *Arabidopsis thaliana* through the floral dip method [51]. Transgenic plants were selected using 2 mg·L^−1^ glufosinate and verified by PCR. T_3_ transgenic lines were generated by self-pollination for subsequent experiments.

### 4.5. Stress Treatments and RNA Extraction

Three different transgenic lines with high expression levels of *PSY* were selected. The wild-type and transgenic seedlings were cultivated on 1/2 MS medium containing 200 mM mannitol or 100 mM NaCl for 14 days under conditions of 26/20 ℃ (day/night), 16/8 h (light/dark), and humidity of 70%. Length of rosette leaf and maximum length of root system of transgenic and WT plants were measured, and 20 biological replicates were performed. The concentration of chlorophyll and carotenoids was extracted in 95% alcohol using the soaking method [52], and three biological replicates were performed. All comparisons in the research were analyzed using one-way ANOVA with Tukey’s test. Significance was defined as *p* < 0.05. Total RNA was isolated from each sample as described above. The first-strand cDNA was used for real-time quantitative RT-PCR (qRT-PCR) analysis. 

### 4.6. Expression Levels of the PSY Gene and Pigment-Related Genes

Quantitative real-time PCR was carried out to measure the expression levels of six pigment-related genes from different samples with the 2^−^^△△^^Ct^ method, following the manufacturer’s procedures of TB Green Premix Ex Taq II (Takara). The AtUBQ10 (NM_116771) gene was used as an internal reference gene for evaluating transcriptional abundance in Arabidopsis [53]. Pigment-related genes include *AtCLH* (AT1G19670.1), *AtNYC* (AT4G13250.1), *AtNOL* (AT5G04900.1), *AtPAL* (AT5G04230), *AtNCED* (AT1G30100), and *AtZDS* (AT3G04870). Relevant primer sequences used are shown in Table S1. All experiments in the research contained three biological replicates.

Quantitative real-time PCR was carried out to measure the expression levels of ZjPSY in different developmental stages and tissues of zoysiagrass and leaves subjected to no-treatment, hormone treatment, drought, or salt stress conditions. The 2-month-old zoysiagrass was inducted with 10 μM GA_3_, 10 μM ABA, 10 μM MeJA, or 0.5 mM SA. Leaves at different growth stages (young, fast-growing, and mature), root tissues and stem tissues were also collected. RNA isolation and cDNA synthesis were performed as above. The *Z. japonica* beta-actin was used as the internal reference gene (GenBank accession no. GU290546) [54]. Relevant primer sequences used are shown in Table S1. All quantitative real-time PCR experiments in the research contained three biological replicates. 

### 4.7. Yeast Two-Hybrid Assay

The ZjPSY ORFs were cloned using the primers pGBKT7-ZjPSY-F/R and inserted into the pGBKT7 vector. The recombinant bait plasmid pGBKT7-ZjPSY was co-transformed into yeast strain Y2H as described in the Yeastmaker™ Yeast Transformation System (Clontech, CA, USA) manufacturer’s instructors. The MATCHMAKER GAL4 yeast two-hybrid system was utilized according to the Match7maker™ Gold Yeast Two-Hybrid System manufacturer’s instructors (Clontech, CA, USA). Matchmaker Insert Check PCR Mix 2 (Clontech, CA, USA) is designed to be used to analyze cDNA inserts (Table S2) in library prey vectors. 

### 4.8. Subcellular Localization Determination

The YFP plasmid and recombinant plasmid 35S::*ZjPSY*:YFP were introduced into *Agrobacterium tumefaciens* strain EHA105 by direct transformation. The young leaves of tobacco were infiltrated with recombinant Agrobacterium strains as described previously [55]. The visualization of YFP and YFP fusion proteins in leaves was performed using Leica TCS SP 8 confocal microscope.

### 4.9. Bimolecular Fluorescence Complementation (BiFC) Analysis

BiFC assays were conducted to verify the interaction between ZjPSY and ZjJ2. Coding sequences of *ZjPSY* and *ZjJ2* were cloned into YNE and YCE vectors, respectively. The primers YNE-ZjPSY-F/R and YCE-ZjJ2-F/R were used. YNE and YCE were transiently expressed in tobacco leaves as described previously [56]. YFP fluorescence signals were analyzed with Leica TCS SP 8 confocal microscope.

## Data Availability

The data presented in this study are available in article or Supplementary Materials.

## Supplementary Materials

The following supporting information can be downloaded at: https://www.mdpi.com/article/10.3390/plants11030395/s1, Figure S1: Sequence analysis of ZjPSY. (A) Intron/Exon organization in the ZjPSY genes. Introns and exons are shown as black lines and yellow boxes, respectively. (B) Secondary structure analysis of ZjPSY protein. Green helix, alpha helix; Blue arrow, beta strand; Faint lines, coil; SS confidence line, the prediction confidence. (C) Protein structure simulation of ZjPSY.; Figure S2: Performance of wild-type and transgenic plants. The transgenic and wild-type Arabidopsis grown in MS agar plates for 14 days (A). 500 mM mannitol (B) and 200 mM Nacl (C) were added to MS medium in drought treatment and salt treatment groups, respectively; Table S1: Primers used in the study; Table S2: cDNA inserts in library prey vectors.
