# Supplementary material for: Expression of ZjPSY, a Phytoene Synthase Gene from Zoysia japonica Affects Plant Height and Photosynthetic Pigment Contents
Source: Plants (Basel). 2022 Jan 31;11(3):395. doi: 10.3390/plants11030395 (PMC8840084; doi:10.3390/plants11030395)
Supplement: Supplementary file 1 [file plants-11-00395-s001.zip › plants-1508006-supplementary.pdf]

Supplementary Table S1. Primers used in the study

Primer name	Primer sequence (5′–3′)
ArUBQ 10-F	GGGCCTTGTATAATCCCTGAT
ArUBQ 10-R	CCATCCTTAGAACCCAACAGC
PSY-qPCR-F2	TTTTGCTGGACGACCTTATGACA
PSY-qPCR-R2	AACCCGACAGTTCCTGCGACAT
ZjPSY-F	GGTACACGACAGTAAGCGCATAC
ZjPSY-R	ACTGCGTACTCCTTCCTTCTATCTA
pGBKT7-ZjPSY-F	ATCCCCGGGAATTCGGCCTCTTAGCTCTGGCTATTTCTC
pGBKT7-ZjPSY-R	AGGAGGACCTGCATATGGCCGCTTTTCTTCCCCCTTGCGG
3302Y-ZjPSY-F	CACGGGGGACTCTTGACCATGGTATTAGCTCTGGCTATTTCTC
3302Y-ZjPSY-R	GGTACACGCGTACTAGTCAGATCGCTTTTCTTCCCCCTTGCGG
YNE-ZjPSY-F	TCGATAGTACTGTCGACCTCATGGCCATCATGCTTGTAAG
YNE-ZjPSY-R	ATCCCGGGAGCGGTACCCTCGCTCTGGCTATTTCTCAGTG
YCE-ZjJ2-F	ACCTCGAGGGTACCGCTCCCATGGTACCAGATTACGCTCATA T
YCE-ZjJ2-R	ACATCGTATGGGTACATCCCAGGGTTTGACTTGATGAGAA
ArCLH1-RT-F	CGCAACATTAGACCCATCCA
ArCLH1-RT-R	GGCTTTCGTCGCCTTACACT
ArNYC-RT-F	CACCTCTTTCTTTCTTCGTCCTA
ArNYC-RT-R	CAATCTTGACCCGACCCGTAT
ArNOL-RT-F	TTTGCTGTTATGGCTGTTCCT '
ArNOL-RT-R	AGTTCCCTCACATCCTTCCCT'
ArPAL-RT-F	ATAAGAGCAGCGACTAAGATGA
ArPAL-RT-R	GGTAACCCGTTGTTGTAGAAAT
ArNCED-RT-F	CGGAGACAGACGAGGTTGTAG
ArNCED-RT-R	TGAGACTTTAGGCCACGGTTC
ArZDS-RT-F	AGTCACTTGGTCGTCGGTTGT '
ArZDS-RT-R	AATTTGGAGGCTGTTCTTGCT
ZjACT-F	GGTCCTCTTCCAGCCATCCTTC
ZjACT-R	GTGCAAGGGCAGTGATCTCCTTG

The efficiency of primer pair PSY-qPCR-F2/R2 is 90.0135，R2=0.9997；ArCLH1-RT-F/R, 104.2102%, R2=0.999; ArNYC-RT-F/R, 102.1831%, R2=0.9998; ArNOL-RT-F/R, 90.4131%, R2=0.9996; ArPAL-RT-F/R, R2=86.5855%, 0.9991; ArNCED-RT-F/R, 107.4709%, R2= 0.9863; ArZDS-RT-F/R, 93.8269%, R2=0.9955.

Supplementary Table S2. cDNA inserts in library prey vectors.

Serial number	Sample	Growth Condition	Time of growth
1	The whole plant	Pots	1 mouth, 3 months, 6 mouths
2	The whole plant	MS plates with 200 mM Nacl	1 mouth
3	The whole plant	MS plates with 500 mM mannitol	1 mouth
4	The whole plant	MS plates with 10 μM GA3	1 mouth
5	The whole plant	MS plates with 10μM ABA	1 mouth
6	The whole plant	MS plates with 10 μM MeJA	1 mouth
7	The whole plant	MS plates with 0.5 mM SA	1 mouth
8	The whole plant	MS plates and MS plates without light	MS plates for 1 mouth and MS plates without light for 5d.
9	All cDNA mentioned in paragraph 4.6		

Total RNA was isolated from each sample as described in paragraph 4.2. Growth Condition.

All pots were kept at 28/23℃ (day/night) with 16h (at 400 mmol/m2/s)/8h photoperiod and 50% humidity.

All Murashige and Skoog plates (4.43 g/L Murashige and Skoog powder, 8 g/L agar, pH 5.8) were kept with a 16h white light (at 90 mmol/m2/s)/8h dark cycle and 50% humidity at 25°C. Shade-treated MS culture bottles are wrapped in tin foil.

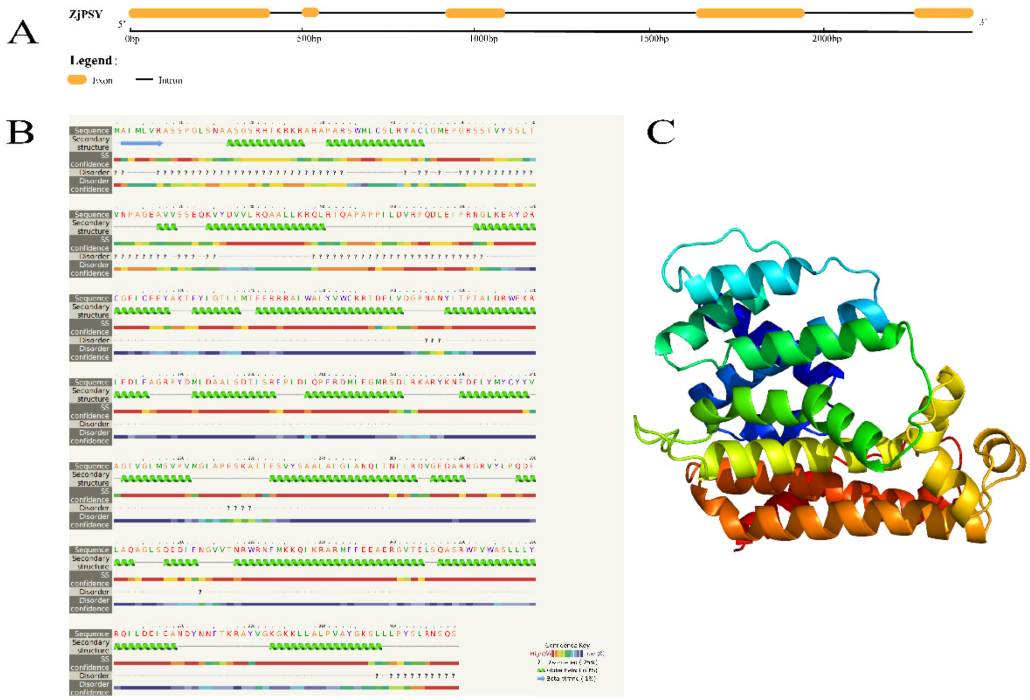

Supplementary Figure S1. Sequence analysis of ZjPSY. (**A**) Intron/Exon organization in the *ZjPSY* genes. Introns and exons are shown as black lines and yellow boxes, respectively. (**B**) Secondary structure analysis of ZjPSY protein. Green helix, alpha helix; Blue arrow, beta strand; Faint lines, coil; SS confidence line, the prediction confidence. (**C**) Protein structure simulation of ZjPSY.

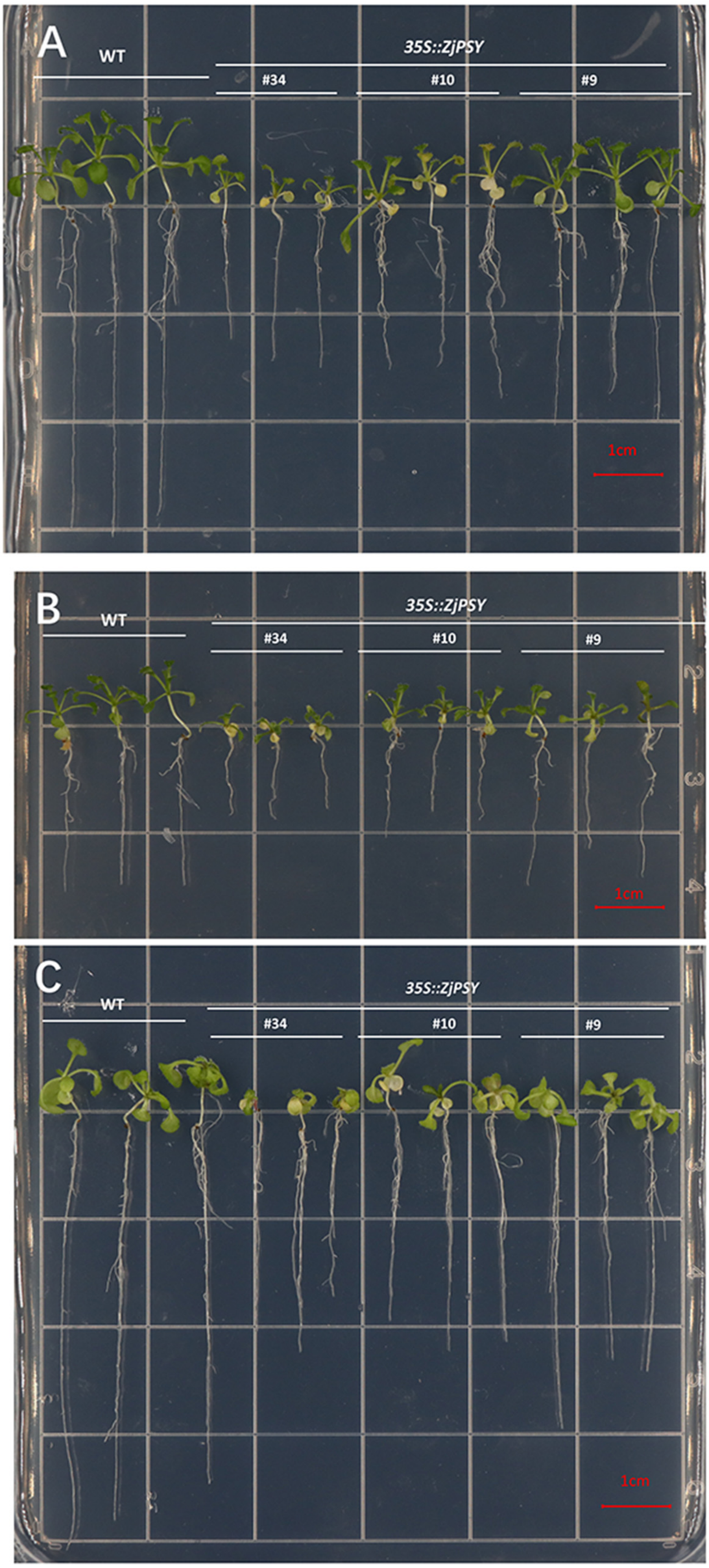

Supplementary Figure S2. Performance of wild-type and transgenic plants. The transgenic and wild-type Arabidopsis grown in MS agar plates for 14 days (**A**). 500 mM mannitol (**B**) and 200 mM Nacl (**C**) were added to MS medium in drought treatment and salt treatment groups, respectively.
